# Long-term safety and efficacy of antithymocyte globulin induction: use of integrated national registry data to achieve ten-year follow-up of 10-10 Study participants

**DOI:** 10.1186/s13063-015-0891-y

**Published:** 2015-08-19

**Authors:** Krista L. Lentine, Mark A. Schnitzler, Huiling Xiao, Daniel C. Brennan

**Affiliations:** Center for Outcomes Research, Saint Louis University School of Medicine, St. Louis, MO USA; Abdominal Transplantation, Saint Louis University School of Medicine, St. Louis, MO USA; Transplant Nephrology, Department of Medicine, Washington University School of Medicine, St. Louis, MO USA; Saint Louis University, Salus Center 4th Floor, 3545 Lafayette Avenue, St. Louis, MO 63104 USA

**Keywords:** Acute rejection, Basiliximab, Clinical trial, Delayed graft function, Kidney transplantation, Induction immunosuppression, Outcome assessment, Mortality, Rabbit antithymocyte globulin, Registries

## Abstract

**Background:**

Rabbit antithymocyte globulin (rATG, Thymoglobulin®) is the most common induction immunosuppression therapy in kidney transplantation. We applied a database integration strategy to capture and compare long-term (10-year) outcome data for US participants in a clinical trial of rATG versus FDA-approved basiliximab.

**Methods:**

Records for US participants in an international, 1-year, randomized clinical trial comparing rATG and basiliximab induction in deceased donor kidney transplantation were integrated with records from the US national Organ Procurement and Transplantation (OPTN) registry using center, transplant dates, recipient sex, and birthdates. The OPTN captures center-reported acute rejection, graft failure, death, and cancer events, and incorporates comprehensive death records from the Social Security Death Master File. Ten-year outcomes according to randomized induction regimen were compared by Kaplan–Meier analysis (two-sided *P*). Non-inferiority of rATG was assessed using a one-tailed equivalence test (a-priori equivalence margins of 0–10 %).

**Results:**

Of 183 US trial participants, 89 % (*n* = 163) matched OPTN records exactly; the remainder were matched by extending agreement windows for transplant and birthdates. Matches were validated by donor and recipient blood types. By Kaplan–Meier analysis, 10 years post-transplant, freedom from acute rejection, graft failure, or death was 32.6 % and 24.0 % in the rATG and basiliximab arms, respectively (*P* = 0.09). The incidence of acute rejection with rATG versus basiliximab induction was 21.0 % versus 32.8 % (*P* = 0.07). Patient survival (52.5 % versus 52.2 %, *P* = 0.92) and graft survival (34.3 % versus 30.9 %, *P* = 0.56) rates were numerically and statistically similar for both arms. Comparison of the composite outcome meets non-inferiority criteria even with a 0 % equivalence margin (one-sided *P* = 0.04). With a 10 % equivalence margin, the odds that rATG is no worse than basiliximab for 10-year risk of the composite endpoint are >99 %.

**Conclusions:**

Ten years post-transplant, rATG induction has comparable efficacy and safety to FDA-approved basiliximab. Integration of clinical trial records with national registry data can enable long-term monitoring of trial participants in transplantation, circumventing logistical and cost barriers of extended follow-up.

**Trial registration:**

ClinicalTrials.gov NCT00235300

**Electronic supplementary material:**

The online version of this article (doi:10.1186/s13063-015-0891-y) contains supplementary material, which is available to authorized users.

## Background

Antibody induction therapy in kidney transplantation is highly effective in reducing acute rejection [1] and, ultimately, in preserving allograft function [2]. Use of induction agents has increased over recent years such that, by 2013, more than 89 % of kidney transplants in the USA were performed with induction therapy [3]. Notably, while rabbit antithymocyte globulin (rATG) is the most commonly used induction agent in contemporary kidney transplantation [3, 4], it was approved by the US Food and Drug Administration (FDA) in 1998 for the treatment of acute rejection but not for the prevention of rejection (induction) [5]. To date only basiliximab and daclizumab, both antibodies against the inter-leukin-2 receptor (IL2R Abs), have been approved by the FDA for use as induction agents in kidney transplantation, and daclizumab is no longer marketed.

A pivotal component of the evidence base informing current understanding of the effectiveness of alternative induction regimens in kidney transplantation came from the 10-10 Study, which was the first prospective, randomized, international clinical trial comparing rATG and basiliximab among deceased donor transplant recipients perceived to be at increased risk of acute rejection or delayed graft function (defined as the need for dialysis within 7 days of transplantation) [6]. The 278 trial participants included 183 patients enrolled at 17 US centers; participants received cyclosporine, mycophenolate mofetil, and prednisone for maintenance immunosuppression. The 12-month study demonstrated that both induction agents were equally effective (*P* = 0.34) in preventing the composite quadruple endpoint of acute rejection, delayed graft function, graft loss, or death. However, when analyzed as a more traditional FDA endpoint of acute rejection, graft failure, or death, the differences were statistically significant in favor of rATG (*P* = 0.02), driven by a lower acute rejection rate in the rATG group (15.6 % versus 25.5 %, *P* = 0.02) [6].

The duration of follow-up in clinical trials is limited by the willingness of patients to participate for extended periods as well as by the ability of investigators to commit the time and resources necessary to track and monitor participants over a number of years. Extended monitoring beyond an initially determined study period may require additional informed consent and always incurs added costs. Consequently, long-term safety and efficacy data are lacking for many drugs in multiple treatment domains, including transplantation [7]. Thus, there is a need for approaches to assessing long-term outcomes for non-FDA-approved drug uses.

Solid organ transplantation is unique among medical specialties in the universal collection of clinical data in national registries in some countries. In the USA, through the mechanism of the Organ Procurement and Transplantation Network (OPTN), as mandated by the National Organ Transplant Act, transplant centers have been required to submit baseline and follow-up clinical data describing all patients listed for and receiving solid organ transplants since 1987 [8]. The OPTN supplements program-reported outcomes information with data from a national death registry, providing a high level of accuracy for the ascertainment of patient and allograft survival [9]. However, owing to a lack of granularity in the collection of baseline information relevant to eligibility and balanced comparisons as required within a clinical trial framework, it has been difficult to draw unbiased inferences on the long-term efficacy and safety of different immunosuppressive regimens based on registry data alone.

Integration of clinical trial and transplant registry records may circumvent some of the logistical difficulties in conducting long-term clinical studies and the limitations of isolated registry analyses. However, despite the opportunity created by the presence of national transplant registries, examples of the use of this approach in transplantation are limited. We previously linked data from the 10-10 Study with OPTN records to assess 5-year clinical outcomes of US-enrolled participants and found that patients treated with rATG had a lower incidence of a traditional composite endpoint of acute rejection, graft failure or death (37 % versus 51 %, *P* = 0.04) [10]. Using a similar approach, 15-year follow-up of 133 Australian participants in the Tricontinental Mycophenolate Mofetil Renal Transplantation Study was recently successfully achieved by linking trial records with follow-up reports from the Australia and New Zealand Dialysis and Transplant Registry (ANZDATA) [11].

Ten years have now passed since completion of enrollment in the rATG versus basiliximab induction immunosuppression trial. The goal of the current study was to link records for US participants in the 10-10 Study with current OPTN follow-up records to compare long-term efficacy and safety over 10 years after transplantation.

## Methods

### Data sources and sampling

Clinical trial data were obtained from a randomized, multi-center international trial involving 278 kidney transplant recipients in the USA and Europe (the 10-10 Study, ClinicalTrials.gov NCT00235300) [6]. The clinical trial compared 1-year post-transplant outcomes after treatment with rATG (*n* = 141) or basiliximab (*n* = 137) as part of immunosuppression regimens in deceased donor renal allograft recipients. In all, 66 % of total participants (*n* = 183) were enrolled at 17 US centers between May 2000 and March 2002. Patient eligibility for trial enrollment was based on prolonged cold ischemia times and donor and recipient risk factors for acute rejection, or delayed graft function, as previously described [6].

Ten-year post-transplant follow-up data for US-enrolled trial participants were obtained from OPTN registry data. As mandated by the National Organ Transplant Act, the OPTN collects information on all solid organ transplant recipients and donors in the USA as submitted by OPTN member centers, including transplant date and baseline demographic and clinical information. Post-transplant follow-up information is collected 6 months after transplant, on the first transplant anniversary, and then annually. Center-reported death records are supplemented within the OPTN registry by the national Social Security Death Master File (SSDMF). The Health Resources and Services Administration (HRSA), of the US Department of Health and Human Services, provides oversight to the activities of the OPTN contractor.

Corresponding OPTN records for trial participants were first sought using transplant center, sex, and exact recorded dates of transplantation and birth in each data source. As the available trial records provided dates of administration of anesthesia for transplant surgery and transplant surgery may occur overnight, OPTN records for trial participants who were not exactly matched initially were sought using trial-reported surgical anesthesia dates ± 1 day. To account for transcription errors, matches were identified for remaining patients using age instead of month and day of birth, or transplant year instead of month and day of transplant. Final matches were validated by comparing trial versus OPTN reports of donor and recipient ABO blood types.

### Approvals

This study was approved by the Saint Louis University Institutional Review Board (protocol number 23175), and by OPTN/HRSA. Because of the anonymity of the patients studied, and the non-intrusive nature of the current research using previously collected records without patient contacts, a waiver of informed consent was granted per the Department of Health and Human Services Code of Federal Regulations (Title 45, Part 46, Paragraph 46.116). Analyses were performed using limited datasets, compliant with the US Health Information Portability and Accountability Act.

### Treatment regimens in the randomized trial

Immunosuppression treatment regimens and regimen allocation used in the randomized trial have been reported previously [6]. Briefly, eligible, enrolled participants were randomized (1:1) to receive either rATG (Thymoglobulin®, Genzyme, Cambridge, MA) a lymphocyte-depleting polyclonal antibody targeting multiple immunologic epitopes, or basiliximab (Simulect®, Novartis Pharmaceuticals) a non-lymphocyte-depleting monoclonal antibody targeting the interleukin-2 receptor for induction immunosuppression. rATG was administered at 1.5 mg per kg of body weight per day intravenously for a total target dose of 7.5 mg per kg of body weight. The first dose was administered intra-operatively prior to graft reperfusion. Basiliximab was administered at 20 mg intravenously on the day of transplant prior to graft reperfusion and on day 4 post-transplant. All patients received cyclosporine (modified), mycophenolate mofetil, and prednisone. Mycophenolate mofetil was initiated prior to transplantation, and the initial dose of cyclosporine was administered by day 4. Investigators used corticosteroid tapering to reduce prednisone to 5 mg per day by 6 months or earlier. Appropriate infection prophylaxis was mandated by the study protocol, as described previously [6].

### Outcomes and ascertainment

The 10-year primary efficacy endpoint was the FDA-specified composite triple endpoint of allograft rejection, graft failure, or patient death used in clinical trials of immunosuppression efficacy. Secondary endpoints included acute rejection, death-censored graft failure, and all-cause graft loss, evaluated individually at 10 years post-transplant. Trial-reported events within the 1-year trial period were supplemented with OPTN records to ascertain long-term outcomes. The OPTN queries centers for information on acute rejection according to periods covered by specific reporting forms (0 to 6 months, 7 to 12 months, then annual periods), but dates of acute rejection within reporting periods are not collected. We defined acute rejection from OPTN records according to center reports that an acute rejection event occurred, taking the event date as the follow-up record date, as per prior methods for identifying acute rejection from OPTN registry data [12, 13]. Acute rejection as a cause of graft failure was included.

Mortality was defined as date of death from any cause, as reported within the trial or from OPTN records based on transplant center or the SSDMF reports. Ascertainment of death, including national death registry SSDMF records, was considered complete. Graft failure was defined as the earliest reported date of return to maintenance dialysis or ‘preemptive’ re-transplantation. Times to acute rejection, graft failure, and the composite triple endpoint were right censored at 10 years post-transplant or center-reported loss-to-follow-up.

Renal function at annual follow-up was quantified as estimated glomerular filtration rate according to the abbreviated Modification of Diet in Renal Disease equation [14]. Serum creatinine values were drawn from OPTN recipient follow-up reporting forms, and age for glomerular filtration rate estimation was computed as age at follow-up.

Diagnoses of malignancy, as captured in the trial reports for up to 1 year post-transplant and by OPTN follow-up reports to 10 years, were examined as a safety endpoint. Time-to-malignancy was computed as the time to the date of first reported cancer diagnosis, and also sub-categorized as time to first reported post-transplant lymphoproliferative disorder, non-melanoma skin cancer or other cancer diagnosis.

### Statistical analyses

Data management and analyses were performed with SAS for Windows software, version 9.3 (SAS Institute Inc., Cary, NC). Distributions of baseline recipient, donor, and transplant factors according to induction treatment assignment, including missing values, were compared using Fisher’s exact test for categorical factors and the Wilcoxon–Mann–Whitney test or the *t* test for continuous variables, as previously reported [6, 10]. No imputation for missing data was performed. The Kaplan–Meier method was used to estimate the frequencies of acute rejection, graft failure, death, or cancer, as well as the primary composite endpoint, at 10 years post-transplant, and the log rank test was applied to compare differences in event frequencies according to induction regimen using a 2-sided *P*-value, to assess superiority. The primary analyses compared outcomes at 10 years post-transplant. Non-inferiority of rATG compared with FDA-approved basiliximab for the composite outcome was assessed using a one-tailed equivalence test with α equal to 0.05 and a-priori defined equivalence margins of 0 % and 10 % [15].

As the prior 5-year linkage study used data extracted shortly after 5 years after trial enrollment [10], and because the OPTN registry incorporates events attributable to prior periods if newly reported by centers after an annual survey, we also re-analyzed 5-year outcomes in the recently extracted data as a secondary analysis. Estimated glomerular filtration rate among survivors with graft function in the two treatment groups was compared at each annual follow-up time by *t* tests.

## Results

### Matching US trial and OPTN registry records

**Fig. 1:**
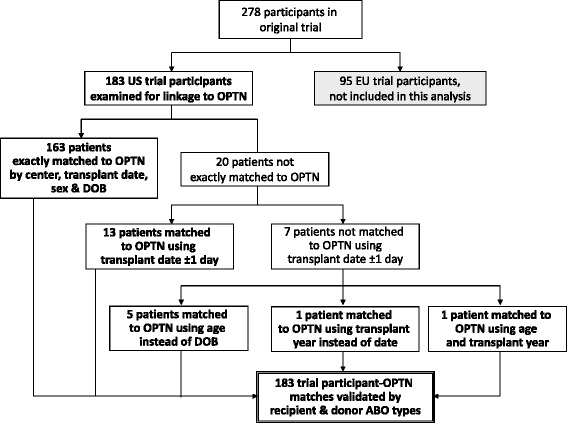
Schematic of data linking procedures and resulting patient matches. DOB, date of birth; OPTN, Organ Procurement and Transplantation Network

Results of the procedures for identifying OPTN reports for US-enrolled trial participants are shown in Fig. 1. A total of 89 % (*n* = 163) of patients were exactly matched in both data sources using center, transplant date, sex, race, and date of birth. Recorded transplant surgery or anesthesia dates varied within ± 1 day for 13 patients (7.1 %) who matched exactly on other parameters. Five (2.7 %) patients were matched using age instead of month and day of birth, along with exact matching for the other parameters. Finally, one (0.5 %) patient was matched using transplant year instead of transplant date and one (0.5 %) final match was identified using age and transplant year along with sex and transplant center. The final 183 matches were validated by recipient and donor ABO types in the trial and OPTN records. Additional file 1 displays the percentage agreement of trial and OPTN records for the matching and validation variables.

### Patient characteristics

As previously reported, 91 of the 183 US participants were randomized to receive rATG induction in the clinical trial and 92 were assigned to receive basiliximab [6]. Baseline recipient and donor characteristics were complete except for cold ischemia time, for which the distribution of missing values was similar across the treatment arms (Table 1). Baseline characteristics were similar among US participants in the two induction groups (Table 1), as previously summarized [10].

**Table 1:** Baseline characteristics of US-enrolled participants in the randomized trial of rATG versus basiliximab induction therapy

	rATG (*n* = 91)	Basiliximab (*n* = 92)	*P*
Recipient characteristics			
Age (years), mean ± standard deviation	49.6 ± 12.4	49.0 ± 13.0	0.73
Male, %	58 %	65 %	0.36
Race, %:			0.29
White	40 %	53 %	
Black	45 %	40 %	
Other	15 %	8 %	
Peak panel-reactive antibody level, mean ± standard deviation	18.7 ± 31.8	17.7 ± 30.7	0.83
Donor characteristics			
Age (years), mean ± standard deviation	42.9 ± 16.3	43.4 ± 17.7	0.84
Donors older than 50 years, %	44 %	47 %	0.77
Male, %	49 %	58 %	0.30
Race, %:			0.80
White	79 %	80 %	
Black	12 %	10 %	
Other	9 %	10 %	
Donation after cardiac death, %	7 %	7 %	0.99
Diabetes mellitus, %	5 %	3 %	0.50
Hypertension, %	30 %	26 %	0.62
Transplant characteristics			
Cold ischemia time (hours), mean ± standard deviation	25.7 ± 9.6	26.0 ± 8.4	0.82
Missing, %	8.8 %	7.6 %	0.77
Previous transplantation, %	5 %	5 %	1.00

From: N Engl J Med, Brennan DC and Schnitzler MA, Long-Term Results of Rabbit Antithymocyte Globulin and Basiliximab Induction, Volume 359, Pages 1736–8, 2008. Massachusetts Medical Society [10]. Reprinted with permission

### Acute rejection, graft survival, and patient survival

US participants randomized to receive rATG had higher freedom from the triple endpoint of rejection, graft failure, or death at 1 year post-transplant (80.0 % versus 68.5 %, two-sided *P* = 0.04) (Table 2). Similar to results detected using OPTN records previously extracted in 2007 [10], significantly higher freedom from the composite endpoint at 5 years post-transplant in the rATG versus basiliximab arm was replicated using the current, updated data (57.6 % versus 44.2 %, 2-sided *P* = 0.04).

**Table 2:** Clinical outcomes among US-enrolled induction therapy trial participants based on integrated trial records and OPTN follow-up data

	rATG %	Basiliximab %	*P*
Outcomes at 1 year			
Acute rejection	14.3 %	22.8 %	0.08
Patient survival	94.5 %	95.7 %	0.74
All-cause graft survival	89.0 %	85.9 %	0.47
Freedom from acute rejection, graft failure or death	80.0 %	68.5 %	0.05
Any malignancy	1.1 %	1.1 %	0.99
Post-transplant lymphoproliferative disorder	0	0	1.00
Non-melanoma skin cancer	1.1 %	0	0.31
Non-skin cancer	0	1.1 %	0.32
Outcomes at 5 years			
Acute rejection	21.0 %	32.8 %	0.07
Patient survival	72.5 %	75.0 %	0.64
All-cause graft survival	67.5 %	60.6 %	0.32
Freedom from acute rejection, graft failure, or death	57.6 %	44.2 %	0.04
Any malignancy	4.5 %	4.5 %	0.97
Post-transplant lymphoproliferative disorder	1.1 %	0	0.31
Non-melanoma skin cancer	2.3 %	1.1 %	0.54
Non-skin cancer	2.3 %	3.3 %	0.67
Outcomes at 10 years			
Acute rejection	21.0 %	32.8 %	0.07
Patient survival	52.8 %	52.2 %	0.92
All-cause graft survival	34.3 %	30.9 %	0.56
Freedom from acute rejection, graft failure, or death	32.6 %	24.0 %	0.09
Any malignancy	9.5 %	8.1 %	0.75
Post-transplant lymphoproliferative disorder	2.2 %	0	0.15
Non-melanoma skin cancer	3.6 %	1.1 %	0.30
Non-skin cancer	6.0 %	6.9 %	0.79

**Fig. 2:**
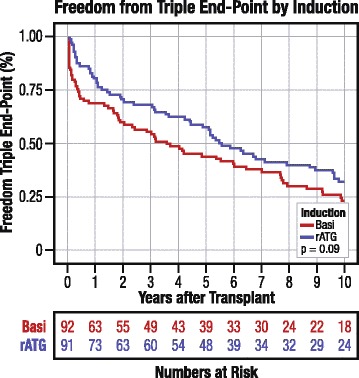
Freedom from triple endpoint according to induction regimen. Basi, basiliximab; rATG, rabbit antithymocyte globulin

By Kaplan–Meier analysis, at 10 years post-transplant, freedom from a composite of acute rejection, graft failure or death was 32.6 % and 24.0 % in the rATG and basiliximab arms, respectively (two-sided *P* = 0.09) (Fig. 2). Comparison of the composite outcome meets non-inferiority criteria even with a 0 % equivalence margin (one-sided *P* = 0.04). With a typical 10 % equivalence margin, the odds that rATG is no worse than basiliximab for 10-year risk of the composite endpoint are greater than 99 % based on the results of this study.

**Fig. 3:**
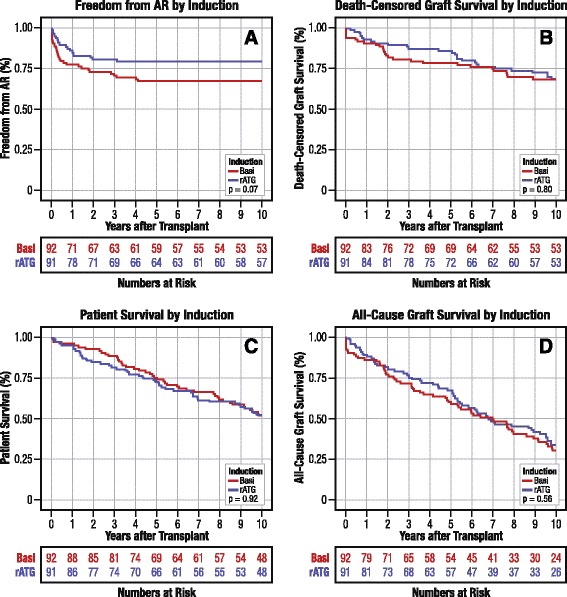
10-year outcomes according to induction regimen. **a** Freedom from acute rejection. **b** Patient survival. **c** Death-censored graft survival. **d** All-cause graft survival. Basi, basiliximab; rATG, rabbit antithymocyte globulin

At 1 year, patients treated with rATG had a non-significant trend towards lower acute rejection incidence (14.3 % versus 22.8 %, two-sided *P* = 0.08) (Table 2). At 5 years post-transplant, the cumulative incidence of acute rejection trended lower among patients randomized to rATG compared with basiliximab, but did not reach significance (21.0 % versus 32.8 %, respectively, two-sided *P* = 0.07). No patients developed incident registry-reported rejection events after 5 years, such that the 10-year incidence of acute rejection was also 21.0 % in the rATG arm and 32.8 % in the basiliximab arm (two-sided *P* = 0.07) (Fig. 3a).

Patient survival was numerically and statistically similar in both treatment groups at 5 years and equivalent at 10 years (rATG, 52.8 %; basiliximab, 52.2 %; *P* = 0.92) (Fig. 3b). Death-censored graft survival was also equivalent in the two groups by 10 years (rATG, 68.5 %; basiliximab, 68.4 %; two-sided *P* = 0.80) (Fig. 3c). Combining trends in mortality and graft failure, all-cause graft survival was generally similar over time among participants randomized to both trial arms, and by 10 years was 34.3 % in those treated with rATG versus 30.9 % in those treated with basiliximab at (two-sided *P* = 0.56) (Fig. 3d).

### Renal function

**Fig. 4:**
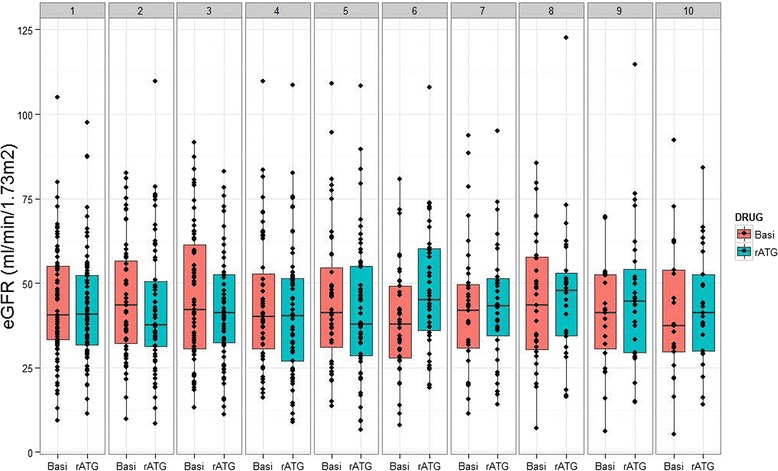
Estimated glomerular filtration rate by trial arm at each annual follow-up time (years post-transplant) among patients with a reported serum creatinine value on corresponding OPTN follow-up forms. Basi, basiliximab; eGFR, estimated glomerular filtration rate; rATG, rabbit antithymocyte globulin

Estimated glomerular filtration rate was computed at each annual follow-up time among patients with a reported serum creatinine value on corresponding OPTN follow-up forms. An additional file summarizes causes of missing serum creatinine values, including graft loss, death, or missed reporting (Additional file 2). The frequency of missed reporting did not differ by treatment group at any follow-up point. Subject counts for glomerular filtration rate estimation declined from 78 and 75 in the rATG and basiliximab arms, respectively, at 1 year to 25 and 21, respectively, at 10 years. Among survivors with graft function and available serum creatinine values at each follow-up year, mean glomerular filtration rate was similar in participants in the two trial arms (Fig. 4).

### Malignancy

The cumulative incidence of any cancer by 10 years post-transplant was similar (*P* = 0.75) among patients who received rATG or basiliximab in the trial, at 9.5 % and 8.1 %, respectively. The 10-year incidences of skin cancer (3.6 % versus 1.1 %, *P* = 0.30), post-transplant lymphoproliferative disorder (2.2 % versus 0 %, *P* = 0.15) and other cancers (6.0 % versus 6.9 %, *P* = 0.79) also did not differ significantly among patients randomized to rATG compared with basiliximab (Table 2).

## Discussion

Results from the first prospective, randomized, multi-center clinical trial comparing the two leading antibody induction agents in renal transplantation, known as the 10-10 Study, provided valuable information on the short-term safety and efficacy of these induction agents [6]. However, information on long-term outcomes associated with initial choice of antibody induction agent is lacking. We integrated clinical trial records for the US participants in the 10-10 Study with current OPTN follow-up records to compare long-term efficacy and safety over 10 years after transplantation.

In the current analysis, we found that patients treated with rATG had similar long-term patient and graft survival at 10 years post-transplant as patients treated with the FDA-approved induction agent basiliximab. Congruent with previously observed superiority of rATG versus basiliximab induction for the FDA-specified composite triple endpoint of allograft rejection, graft failure or patient death used in clinical trials of immunosuppression efficacy observed at 1 year post-transplant [6, 10], superiority with rATG induction for this composite endpoint was confirmed at 5 years using current data for this US-based sub-analyses. At 10 years post-transplant, while freedom from the composite of acute rejection, graft failure or death was not significantly different among US participants in the two trial arms, non-inferiority for rATG compared with FDA-approved basiliximab for 10-year risk of the composite endpoint was demonstrated with a high level of statistical certainty.

Because rATG is a more potent induction agent than IL2R Abs, there is concern over increased risks of death and malignancy in the long-term. We found that there were no significant differences in these outcomes at 10 years. Although our observations contradict prior reports raising concern for more common post-transplant malignancies with rATG [16, 17], there were numerically higher point estimates of lymphoproliferative disorder and non-melanoma skin cancer, but not other non-skin cancer, in the rATG arm, and the lack of significance may be impacted by the low study power. Continued assessment of the impact of induction immunosuppression on cancer risk is warranted. Importantly, the close alignment of the patient survival curves and very similar magnitude of the 10-year survival fractions supports truly equivalent long-term survival. A strength of the current analysis is that we studied a carefully characterized patient sample with confirmed induction exposure, and linked to long-term transplant registry data for outcomes ascertainment, including comprehensive national death records. We did not assess early complications aside from those captured as study outcomes in the current analysis. Common concerns with the use of rATG early after transplantation include thrombocytopenia, leucopenia, pyrexia, and hypotension. We previously reported associations of rATG with transient thrombocytopenia and leucopenia (driven by lymphopenia) that resolved by post-operative day 14 [6].

Maintenance immunosuppression warrants consideration in any study of induction therapies. Cyclosporine, mycophenolate, and low-dose prednisone were used in the 10-10 Study. While the most common maintenance immunosuppression regimen in current US practice is tacrolimus, mycophenolate, and low-dose prednisone, cyclosporine continues to be used as the primary calcineurin inhibitor among many patients internationally. A subsequent randomized comparison of rATG versus the IL2R Ab daclizumab in the context of maintenance tacrolimus, mycophenolate, and low-dose prednisone among high immunologic-risk patients showed that rATG induction was associated with lower rates of delayed graft function (32 % versus 45 %, *P* = 0.04), acute rejection (15 % versus 27 %, *P* = 0.02), and need for anti-lymphocyte treatment of acute rejection (2.7 % versus 14.9 %, *P* = 0.002) at 1 year post-transplant [18]. This benefit of rATG in lowering acute rejection compared with an IL2R Ab was nearly identical to the benefit seen in the 10-10 Study, despite the use of tacrolimus as the maintenance calcineurin inhibitor. Furthermore, a recent open-label trial comparing rATG versus dacluzimab or basiliximab, also in the context of maintenance tacrolimus, mycophenolate, and corticosteroids, found that only rATG was protective against 6-month and 1-year acute rejection in African-American recipients [19]. Finally, our analysis considered maintenance therapy from an intention-to-treat perspective, but it is possible that maintenance regimens changed over time.

There are other limitations to our study. We were unable to capture follow-up data for the European patients enrolled in the original trial, owing to use of a US-based transplant registry. No similar comprehensive registry existed in the participating European countries at the time of the trial. As a result, 34 % of the initial sample was not included in the current analysis, compromising statistical power. Although 96 % of trial and OPTN records matched exactly on all linkage parameters or with ±1 day extension of transplant date (to account for differences in recorded anesthesia induction versus transplant date in the two sources), 4 % of matches required broadening of agreement windows for transplant or birthdates, and thus indicate recording errors for these dates in one of the sources; such recording errors cannot be resolved with the current design based on extracted information without direct access to center records. Observed baseline characteristics were well balanced across the included subjects in the two treatment arms, but imbalances in unmeasured characteristics are possible. While the intervention of interest, induction immunosuppression, is solely an early exposure, loss of blinding after the completion of the trial and subsequent unmeasured impacts on other aspects of care is also a potential limitation of the study design.

The long-term data presented were also limited by the fields captured in the OPTN registry. For example, histological assessments of the type and severity of acute rejection episodes are not collected by the OPTN. Although center-reported death records were supplemented with the SSDMF and thus considered to be complete, outcomes (acute rejection, graft failure) that relied solely on center reports could be incompletely captured. Because the OPTN registry incorporates events attributable to prior periods if newly reported by centers after an annual survey, we also re-analyzed 5-year outcomes in the recently extracted data and found slightly higher event rates compared with the prior 2007 extraction [10] but with very consistent patterns. It is possible that re-extraction after a longer latency could also impact estimates of 10-year event frequencies if some events close to the end of the study period were recognized and reported after our data extraction.

Conducting clinical trials with long-term follow-up poses many challenges, from financial burdens to the unwillingness of patients, physicians, and commercial and academic sponsors to participate in studies that require years of involvement. Consequently, long-term safety and efficacy data are lacking for most drugs [20]. Although post-marketing surveillance for adverse effects is mandatory by the FDA, the success of such programs relies heavily on spontaneous reporting by healthcare providers. In the field of transplantation in particular, although the majority of contemporary immunosuppressive agents have been used in clinical practice for decades, there are few long-term, prospective clinical trials evaluating safety and efficacy [21–25]; hence, the optimal immunosuppressive regimen remains a subject of debate. Here, we describe an efficient, non-obtrusive method of monitoring long-term treatment outcomes using the combination of a properly designed clinical trial and registry outcomes data. Our data integration approach could be particularly useful in addressing other uncertainties for immunosuppression-related outcomes in transplantation when clinical trial and registry data are available. Despite the opportunity created by the presence of national transplant registries, we are aware of only two prior demonstrations of this approach in transplantation: a 5-year follow-up of the 10-10 Study by our group [10], and linkage of records from a maintenance immunosuppression trial with ANZDATA records [11]. We believe this methodology has potential for more common application in the field of transplantation, as well as broader implications for other fields with access to both trial and registry data.

## Conclusions

In summary, integrating records from a clinical trial of induction immunosuppression therapies with national transplant registry data enabled ascertainment of long-term follow-up information for 9 years beyond the duration of the original comparative trial. We found that, compared with an FDA-approved induction agent, the benefits of polyclonal induction with rATG for an FDA-defined composite endpoint of acute rejection, graft failure, or death were sustained through 5 years post-transplant. At 10 years post-transplant, rATG induction had comparable efficacy and safety to FDA-approved basiliximab. Our methodology demonstrates an efficient strategy for monitoring the long-term safety and efficacy of therapies examined in clinical trials when registry data for participants are also available. This approach may be extended to clinical trials outside of transplantation.

## Additional files

Additional file 1:
**Agreement of variables used for matching and validation of clinical trial and OPTN records.** This file displays the percentage agreement of trial and OPTN records for the matching and validation variables. (PDF 51 kb) Additional file 2:
**Counts of available serum creatinine results from annual OPTN follow-up forms and causes of missing serum creatinine values over 10 years post-transplant.** This file summarizes causes of missing serum creatinine values including graft failure, death, and missed reporting. (DOCX 16 kb)

## Abbreviations

ANZDATA: Australia and New Zealand Dialysis and Transplant Registry
FDA: US Food and Drug Administration
HRSA: Health Resources and Services Administration
OPTN: Organ Procurement and Transplantation Network
rATG: rabbit antithymocyte globulin
SSDMF: Social Security Death Master File
