# Erratum to: Long-term safety and efficacy of antithymocyte globulin induction: use of integrated national registry data to achieve ten-year follow-up of 10–10 Study participants

**DOI:** 10.1186/s13063-015-0940-6

**Published:** 2015-09-16

**Authors:** Krista L. Lentine, Mark A. Schnitzler, Huiling Xiao, Daniel C. Brennan

**Affiliations:** Center for Outcomes Research, Saint Louis University School of Medicine, St. Louis, MO USA; Abdominal Transplantation, Saint Louis University School of Medicine, St. Louis, MO USA; Transplant Nephrology, Department of Medicine, Washington University School of Medicine, St. Louis, MO USA; Saint Louis University, Salus Center 4th Floor, 3545 Lafayette Avenue, St. Louis, MO 63104 USA

After publication of this article, it has come to our attention that a few corrections had not been updated. We are publishing this erratum to highlight what has been updated from the original article. The updates are as follows:-

Abstract – the abstract has been updated to reflect the figures in Table 2, patient survival percentage has been updated from *52.5 %* to *52.8 %*.

Table 2 – The P value for *Freedom from acute rejection, graft failure or death* has been updated from 0.05 to 0.04.

Figure 3 captions were incorrect on the original article. This has now been updated. The caption for 3b was updated from Patient survival to Death-censored graft survival. The caption for 3c was updated from Death censored graft survival to Patient Survival.

The corresponding text relating to Fig. 3 has also been updated in line with the figure changes. In the original article it was:-

*Patient survival was numerically and statistically similar in both treatment groups at 5 years and equivalent at 10 years (rATG, 52.8 %; basiliximab, 52.2 %; P = 0.92) (Fig. 3b). Death-censored graft survival was also equivalent in the two groups by 10 years (rATG, 68.5 %; basiliximab, 68.4 %; two-sided P = 0.80) (Fig. 3c). Combining trends in mortality and graft failure, all-cause graft survival was generally similar over time among participants randomized to both trial arms, and by 10 years was 34.3 % in those treated with rATG versus 30.9 % in those treated with basiliximab at (two-sided P = 0.56) (Fig. 3d)*.

This has now been updated to the following:-

*Death-censored graft survival was also equivalent in the two groups by 10 years (rATG, 68.5 %; basiliximab, 68.4 %; two-sided P = 0.80) (Fig. 3b). Patient survival was numerically and statistically similar in both treatment groups at 5 years and equivalent at 10 years (rATG, 52.8 %; basiliximab, 52.2 %; P = 0.92) (Fig. 3c). Combining trends in mortality and graft failure, all-cause graft survival was generally similar over time among participants randomized to both trial arms, and by 10 years was 34.3 % in those treated with rATG versus 30.9 % in those treated with basiliximab (two-sided P = 0.56) (Fig. 3d)*.
