# Supplementary figures and images for: Long-term safety and efficacy of antithymocyte globulin induction: use of integrated national registry data to achieve ten-year follow-up of 10-10 Study participants
Source: Trials. 2015 Aug 19;16:365. doi: 10.1186/s13063-015-0891-y (PMC4545548; doi:10.1186/s13063-015-0891-y)

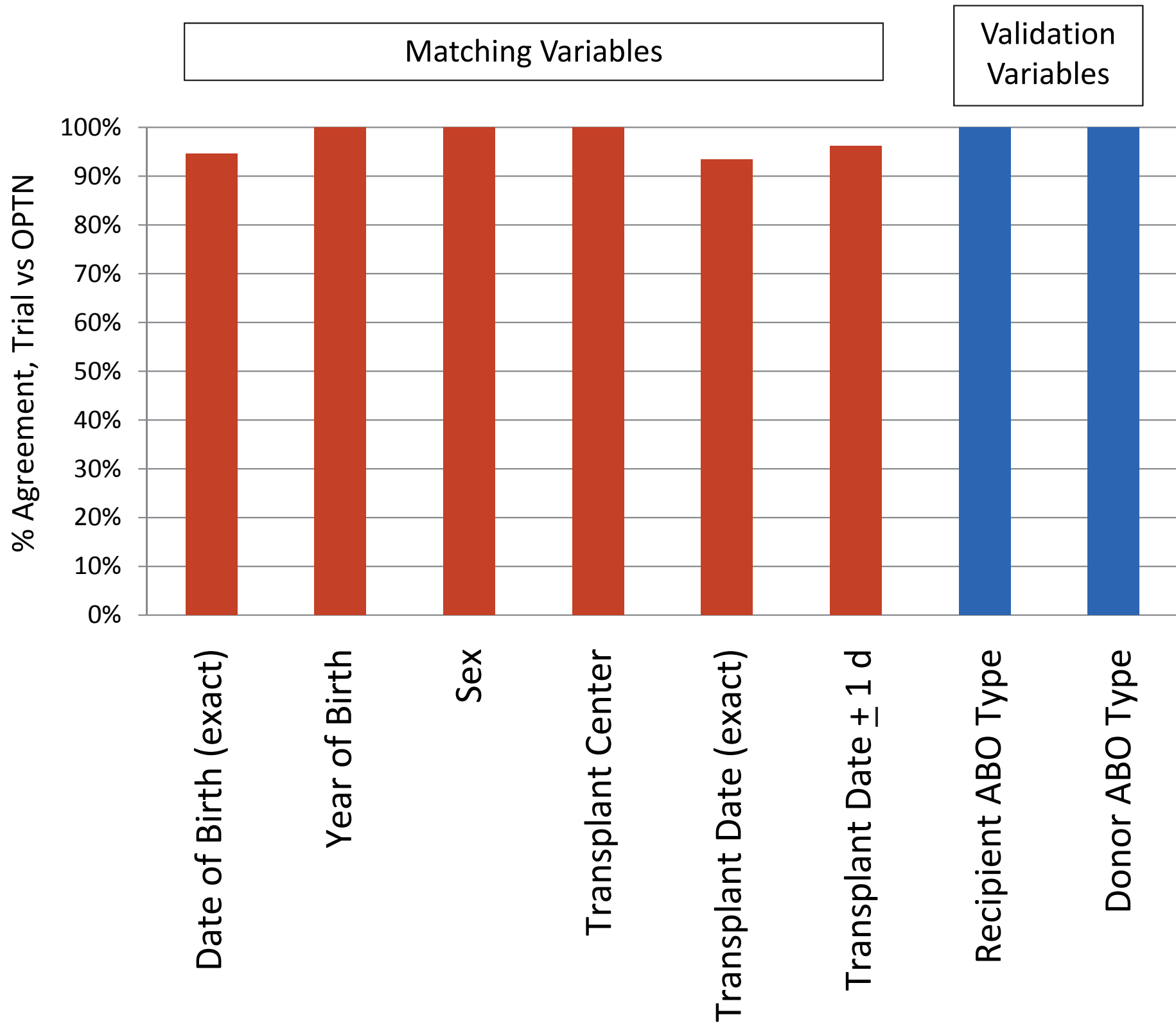

Supplement: Additional file 1: — Agreement of variables used for matching and validation of clinical trial and OPTN records. This file displays the percentage agreement of trial and OPTN records for the matching and validation variables. (PDF 51 kb) [file 13063_2015_891_MOESM1_ESM.pdf]
