# Supplementary material for: Long-term safety and efficacy of antithymocyte globulin induction: use of integrated national registry data to achieve ten-year follow-up of 10-10 Study participants
Source: Trials. 2015 Aug 19;16:365. doi: 10.1186/s13063-015-0891-y (PMC4545548; doi:10.1186/s13063-015-0891-y)
Supplement: Additional file 2: — Counts of available serum creatinine results from annual OPTN follow-up forms and causes of missing serum creatinine values over 10 years post-transplant. This file summarizes causes of missing serum creatinine values including graft failure, death, and missed reporting. (DOCX 16 kb) [file 13063_2015_891_MOESM2_ESM.docx]

**Additional 2, Table**. Counts of available serum creatinine information from annual OPTN follow-up forms and causes of missing serum creatinine values over ten years post-transplant.

|  |  |  |  | **Event as Cause of Missing SCr** | | |
| --- | --- | --- | --- | --- | --- | --- |
| **Year** | **Treatment Group** | **Total N** | **Patients with SCr in Period** | **Graft Failure ^a^** | **Death ^b^** | **Missing Form or SCr Result** |
| 1 | rATG | 91 | 78 | 2 | 4 | 7 |
|  | Basiliximab | 92 | 75 | 2 | 2 | 13 |
| 2 | rATG | 91 | 62 | 5 | 7 | 17 |
|  | Basiliximab | 92 | 59 | 9 | 2 | 22 |
| 3 | rATG | 91 | 45 | 6 | 10 | 30 |
|  | Basiliximab | 92 | 44 | 11 | 7 | 30 |
| 4 | rATG | 91 | 57 | 7 | 12 | 15 |
|  | Basiliximab | 92 | 51 | 12 | 12 | 17 |
| 5 | rATG | 91 | 55 | 9 | 15 | 12 |
|  | Basiliximab | 92 | 44 | 12 | 16 | 20 |
| 6 | rATG | 91 | 43 | 15 | 18 | 15 |
|  | Basiliximab | 92 | 41 | 13 | 19 | 19 |
| 7 | rATG | 91 | 35 | 17 | 21 | 18 |
|  | Basiliximab | 92 | 35 | 15 | 24 | 18 |
| 8 | rATG | 91 | 26 | 18 | 24 | 23 |
|  | Basiliximab | 92 | 31 | 19 | 25 | 17 |
| 9 | rATG | 91 | 31 | 19 | 26 | 15 |
|  | Basiliximab | 92 | 23 | 20 | 29 | 20 |
| 10 | rATG | 91 | 25 | 22 | 29 | 15 |
|  | Basiliximab | 92 | 21 | 20 | 31 | 20 |

^a^ Indicates graft failure in current period and no reported serum creatinine value for the period, or graft loss prior to the period

^b^ Indicates death without non-fatal graft loss in current period and no reported serum creatinine for the period, or death prior to the period.

Chi-square test of Missing Reporting vs Treatment Group: P>0.05 for all years
